# Diffusion Tensor Imaging in Amyotrophic Lateral Sclerosis: Machine Learning for Biomarker Development

**DOI:** 10.3390/ijms24031911

**Published:** 2023-01-18

**Authors:** Anna Behler, Hans-Peter Müller, Albert C. Ludolph, Jan Kassubek

**Affiliations:** 1 Department of Neurology, University of Ulm, Oberer Eselsberg 45, 89081 Ulm, Germany; 2 German Center for Neurodegenerative Diseases (DZNE), 89081 Ulm, Germany

**Keywords:** amyotrophic lateral sclerosis, diffusion tensor imaging, motor neuron disease, magnetic resonance imaging, artificial intelligence, machine learning

## Abstract

Diffusion tensor imaging (DTI) allows the in vivo imaging of pathological white matter alterations, either with unbiased voxel-wise or hypothesis-guided tract-based analysis. Alterations of diffusion metrics are indicative of the cerebral status of patients with amyotrophic lateral sclerosis (ALS) at the individual level. Using machine learning (ML) models to analyze complex and high-dimensional neuroimaging data sets, new opportunities for DTI-based biomarkers in ALS arise. This review aims to summarize how different ML models based on DTI parameters can be used for supervised diagnostic classifications and to provide individualized patient stratification with unsupervised approaches in ALS. To capture the whole spectrum of neuropathological signatures, DTI might be combined with additional modalities, such as structural T1w 3-D MRI in ML models. To further improve the power of ML in ALS and enable the application of deep learning models, standardized DTI protocols and multi-center collaborations are needed to validate multimodal DTI biomarkers. The application of ML models to multiparametric MRI/multimodal DTI-based data sets will enable a detailed assessment of neuropathological signatures in patients with ALS and the development of novel neuroimaging biomarkers that could be used in the clinical workup.

## 1. Introduction

Neuroimaging is a standard tool in the clinical workup of neurodegenerative diseases. Parameterization of imaging data enables the development of objective and reliable biomarkers. A computational analysis of neuroimaging parameters has the potential to provide insights into complex disease mechanisms and might close important gaps in research [1]. As amyotrophic lateral sclerosis (ALS) is characterized by degeneration of the upper and lower motor neurons of the cerebral cortex, brainstem, and spinal cord [2], neuroimaging is a promising tool to assess neuropathology in vivo. The neuropathology of ALS is associated with a regional, four-stage distribution pattern of phosphorylated TDP-43 aggregates, as confirmed in postmortem studies [3,4]. Given that it is well-established that ALS is regarded as a multisystem disorder with extra-motor involvement [5], patients with ALS exhibit significant clinical heterogeneity, particularly in terms of site of onset, rate of progression, and cognitive impairment [6], which makes it difficult to effectively stratify patients for clinical trials in the development of novel therapies. Phenotypic heterogeneity hampers stratifying patients in early disease stages for clinical trials [7].

Magnetic resonance imaging (MRI) is part of the diagnostic procedures of ALS to exclude structural lesions and other etiologies of the clinical presentation. Objective and reliable in vivo neuroimaging biomarkers for early and accurate individualized prognosis and assessment of the cerebral status in ALS are still missing. Diffusion-weighted MRI (DWI) and diffusion tensor imaging (DTI) play a key role in this regard, as this MRI modality can reveal alterations in white matter (WM) by measuring the differences in constraints on the water diffusion in the brain [8]. The widely used scalar fractional anisotropy (FA) quantifies the degree of water diffusion anisotropy within voxels [9]. FA values range between 0 (highly isotropic diffusion) and 1 (highly anisotropic diffusion). Other metrics used to describe the constraints of water diffusion are the mean diffusivity (MD), i.e., the magnitude of the mobility of water molecules independent of directionality, the axial diffusivity (AD), i.e., the magnitude of diffusion parallel to axonal fiber tracts, and radial diffusivity (RD), i.e., diffusion in the direction perpendicular to the axonal fibers [9]. While FA is a summary measure of microstructural integrity and, thus, highly sensitive to microstructural changes, it is less specific to the type of change. MD is an inverse measure of the membrane density, AD tends to be variable in WM changes and axonal injury, and RD increases in WM with dysmyelination. Changes in the axonal diameters or density may also influence RD [10]. At the group level, WM alterations in neurodegenerative diseases can be assessed by unbiased whole-brain-based voxel-wise comparison of DTI metrics (e.g., [11]) or, after fiber tract reconstruction, by hypothesis-guided tract-wise analysis of DTI metrics (e.g., [11]).

In ALS, extensive regional alterations in diffusion metrics in the brain have been demonstrated [12,13]. Consistent findings are reductions in the FA along the corticospinal tract (CST) [14,15] and in the corpus callosum (CC) [16]. Longitudinally, the decrease in FA in the CST is associated with the disease severity [17]; recently, a prospective multicenter study demonstrated the feasibility of this regional diffusion metric as a progression marker [11].

The in vivo analysis of specific WM neuronal tracts allows for the neuropathological staging pattern to be translated to a DTI staging scheme for patients with ALS via a hypothesis-driven tract-of-interest-based approach [11]. During the progression of ALS, microstructural alterations indicated by decreased FA values occurring sequentially in the following tracts: the CST is affected first (stage 1), followed by the corticorubral and corticopontine tracts in stage 2 and the corticostriatal pathway in stage 3, while the involvement of the proximal part of the perforant pathway marks stage 4. The longitudinal applicability of this individualized staging system was confirmed [11].

In the evaluation of new therapeutic approaches in ALS, the current focus is on survival and loss of functionality as endpoints in clinical trials [18]. For example, fluid markers, such as neurofilaments (NF) [19], have proven successful in their use as biomarkers; here, DTI-based neuroimaging could be a valuable addition given that, ideally, a longitudinal biomarker should represent the (regional) progression of neuropathology. However, clinical scores are not fully able to capture cerebral alterations. In addition, functionality might temporarily not deteriorate under the influence of a drug without a change in the actual disease progression. Objective and reliable neuroimaging biomarkers that are sensitive to the progression of neuropathology in vivo might rectify this situation and potentially serve as endpoints in clinical trials.

This review summarizes the advantages and the potential of machine learning (ML)-based DTI methods for patient diagnosis and monitoring, and the future design of clinical DTI applications to ALS is conceptualized. As univariate neuroimaging methods have been successful at the group level but are of restricted usefulness at the individual level in providing definitive clinically useful biomarkers, ML approaches could also be employed for improving individual differential diagnosis.

## 2. Diagnostic Models

**Figure 1:**
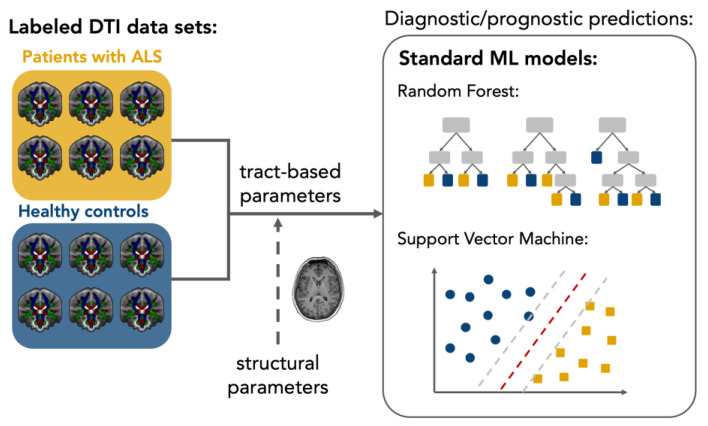
Schematic setup of a diffusion tensor imaging (DTI)-based supervised machine learning application in amyotrophic lateral sclerosis (ALS). Relevant features for classification are extracted from labeled data sets of patients and controls; for example, using tract-based analysis approaches. Standard machine learning models are trained on the labeled data and can subsequently also make diagnostic predictions for new data sets. An extension of the DTI features by parameter structural MRI is feasible.

The clinical relevance of FA alterations along the CST is limited in the light of a meta-analysis of 30 studies which showed a pooled sensitivity of 65% for differentiating between patients with ALS and healthy persons [20]. Sensitivity varied little between studies, although regional FA quantification differed between region of interest (ROI)-based and tractography approaches and different field strengths. However, other regional ALS-associated FA alterations do not outperform the discrimination power of the CST [16,20,21]. Multivariate analysis approaches are promising to overcome the limitations of individually used DTI parameters for diagnostic predictions. Traditional statistical multivariate methods, such as *z*-score approaches, allow quantitative interpretation at the level of the individual subject in ALS [22], but capturing and accounting for the complex interactions between individual parameters remains difficult in these attempts. By analyzing high-dimensional data sets using ML models, novel opportunities are emerging for developing multivariate DTI biomarkers in ALS. As a branch of artificial intelligence (AI), ML algorithms and models automatically extract information from data to identify undiscovered patterns and relationships between different features and provide individualized predictions in healthcare settings [23]. Supervised ML is suitable for a wide range of binary classifications, such as predicting group membership in ‘patient vs. healthy controls’ scenarios, based on biomedical features in ALS research [24]. In clinical neuroimaging with sometimes limited sample sizes, standard supervised models, such as support vector machines (SVMs) and random forests (RFs), have been used in diagnostic settings and demonstrated their usefulness by retrieving robust results [1,25]. SVMs provide a robust classification algorithm by transforming data into a high-dimensional feature space, where a margin is maximized to separate classes [26]. Due to the transformation of the data, the underlying decision-making logic of the SVM cannot be interpreted directly. Decision trees provide better interpretability by classifying based on numerous binary decisions obtained from the data. An RF combines the results of an ensemble of decision trees, which increases the accuracy [27]. Overfitting (or underfitting) of ML algorithms is likely to occur with a limited number of samples per feature, i.e., parameter, in the model processing [24]. A minimum sample-to-feature ratio (SFR) of 10–15 is proposed as necessary based on historical statistical models, but modern algorithms may provide good fitting results with a lower SFR [28]. The implementation of standard ML models on DTI data is illustrated in Figure 1.

In addition to the prediction outcomes, most ML models also determine the importance of the features used, which enables data-driven feature selection. For such an unbiased approach to diagnostic classification in ALS, an SVM was used with all voxels of the FA maps as features (at a sample size of about 20 per group) [29]. Although an accuracy of 83% could be achieved after reducing the features to the 2400~3400 most discriminating ones in a second step, this result should be considered with caution due to the very low SFR and without validation on an independent test sample. Since ALS is a relatively rare disease, the sample size in (monocentric) clinical trials is often limited. Therefore, it might be useful to determine features a priori based on previous results in ALS from group studies and/or neuropathological considerations to address the SFR with the typically limited sample sizes [13]. The CST, therefore, is brought into focus as a predominant finding in group comparisons. Thus, RFs on all four diffusion metrics (FA, MD, RD, and AD) of the voxels assigned to the CST enabled inferences on the overall profile of the CST, leading to a diagnostic accuracy of 80% [30]. An addition of diffusion parameters of motor callosal tracts to CST diffusion metrics achieved a similar accuracy of 78% in predicting patients with SVM, based on the training data set [31]. An overview is presented in Table 1.

**Figure 2:**
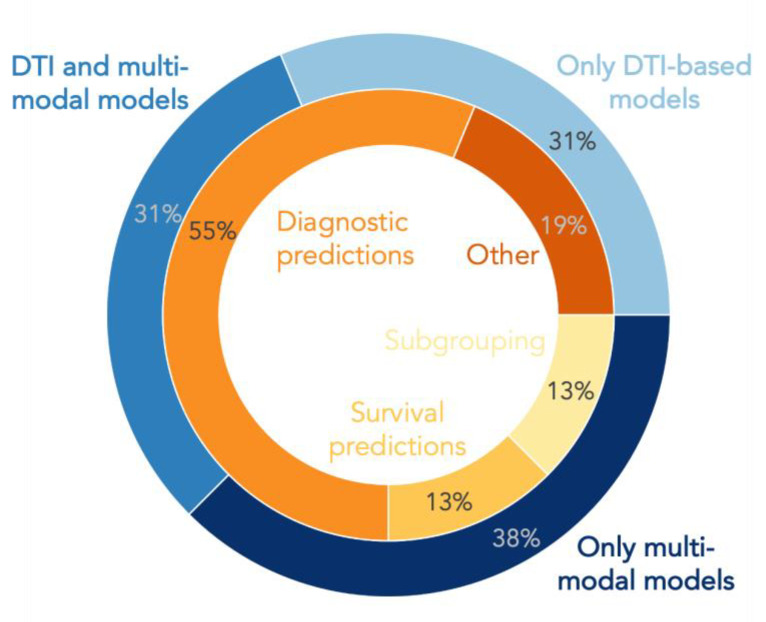
Pie plot displaying the percentage of studies using diffusion tensor imaging (DTI)-based feature sets or combinations of DTI with other modalities in machine learning and deep learning models for different research tasks in amyotrophic lateral sclerosis.

In addition to diagnostic classifications, there are a few other ML applications on DTI data (see Figure 2).

It could be shown by ML model applications to DWI that the WM network can be used as a biomarker to predict the progression rate in ALS patients at the single-subject level [34]. Additional computational algorithms may enable the modeling of sequential processes from cross-sectional data without prior theoretical assumptions about the progression of neuropathology. Thus, event-based modeling identified the CST and the CC as the WM structures first impacted in ALS [36]. This is consistent with the inclusion of the CST as the first stage in the DTI staging scheme [11] and its use in diagnostic ML models. By extending the feature selection for diagnostic predictions with diffusion metrics of the tracts associated with the neuropathological stages 2–4, the accuracy of an SVM could be slightly improved [32]. However, even extending the classification with features associated with advanced disease stages could not overcome the fact that unimodal brain imaging analyses cannot fully assess the complex neuropathology in patients with ALS [13,39]. Indications of restricted diagnostic accuracy of other MRI modalities are also evident in SVM classifications based solely on structural MRI parameters [31,32] or on resting-state functional MRI (fMRI) [40]. Based on multiparametric MRI assessments, feature sets combined from diffusion metrics and structural parameters, such as cortical thickness or texture properties, uniformly lead to an increase in the diagnostic accuracy of SVMs [31,32]. The superiority of such multiparametric MRI feature sets with WM and grey matter (GM) parameters over uniparametric approaches was also evident when a canonical discriminant function [41] or multilayer perceptrons [32,38], i.e., state-of-the-art artificial neural networks, were used for diagnostic classifications. The combination of DTI data with (resting state) fMRI is less studied. Nevertheless, the integration of patients’ FA maps together with their default-mode networks in RF models demonstrated a higher discriminative power for the classification of ALS than feature sets from the two individual MRI modalities [35]. An overview is presented in Table 2.

The neuroimaging signatures of patients with ALS might be confounded by disease duration or gender differences [47]. To address such sources of potential bias in classification models, one study selected anatomical domains as features that showed statistically significant differences in group comparisons between patients and healthy controls after adjustment for age, sex, and disease duration [42]. The multivariate binary logistic regression classifier then achieved a diagnostic accuracy of only 78% in an independent validation sample. Although feature selection based on statistical tests is apparently attractive, it has already been highlighted that high statistical significance does not automatically imply high discriminatory power in ML models [48].

Overall, the combination of DTI parameters with parameters from other MRI modalities significantly increases diagnostic sensitivity. Nevertheless, a perfect identification of all patients with ALS in a group mixed with healthy people seems to remain unachievable solely on DTI metrics.

## 3. Phenotypic Differentiation

Although diagnostic approaches are most important in the use of ML in clinical neuroimaging, the diagnosis of ALS is based on clinical criteria, such as the El Escorial diagnostic criteria [49] and the Gold Coast Criteria [50,51], is well-established for ALS. Still, there are diagnostic pitfalls (especially in patients with so-called mimic disorders [52])—the discussion of the clinical criteria is beyond the scope of this review. In diagnostic classification neuroimaging-based models, the issue of the differentiation of mimic disorders can be addressed by having the model decide not between ‘diseased’ and ‘healthy’ but between different patient categories. RF achieved an accuracy of 87% for ALS vs. clinical mimics based on diffusion features [31]. The revision of the El Escorial diagnostic criteria from 2015 included restricted phenotypes, i.e., primary lateral sclerosis (PLS), flail arm syndrome (FAS), progressive bulbar palsy (PBP), and progressive muscular atrophy (PMA)/lower motor neuron disease (LMND) [53]. However, this concept of the restricted phenotypes of ALS remains discussed [54,55]. With DTI, unbiased, quantitative statements about WM alterations in phenotypes are possible to contribute to these discussions. Fast-progressing LMND, PBP, PLS, and FAS show microstructural alterations whose patterns are identical to ‘classical’ ALS [56,57,58]. In a large-scale study with 575 patients with ALS including different phenotypes, texture properties of all five areas of the CC and diffusion metrics of the associated tracts were investigated and, subsequently, the discriminating value of those CC MRI metrics was evaluated by an SVM [59]. The model trained solely on patients with ‘classical’ ALS was also able to identify patients with different phenotypes with a sensitivity between 80% and 86% in independent test data sets. The results of this unbiased approach support the classification of clinical phenotypes as ALS variants and highlight the discriminating power of CC features which may contribute to further combined neuroimaging markers with high biomarker potential. Clustering algorithms as unsupervised ML models are appropriate to provide insights into similarities of neurodegeneration patterns between patients without prior hypotheses. In particular, clustering models with multimodal feature sets might be a valuable contribution to dissecting the heterogeneity of ALS. Thus, based on multiparametric MRI features obtained from structural MRI and DTI, a probabilistic network-based clustering algorithm reliably divided patients into three clusters with similar patterns of cerebral involvement [33]; these three clusters could be interpreted as patient subgroups. Across clusters, patients showed distinct clinical features and cognitive profiles. Therefore, each cluster might indicate a different neuroimaging phenotype of ALS which can be described as a pure motor, a frontotemporal, and a cingulate-parietal-temporal variant of ALS [45]. In the context of ALS phenotypes and variants, neuroimaging-based ML models might potentially be an effective tool to stratify patients aside from ‘classical’ ALS for clinical trials.

For individualized patient stratification in ALS, models predicting disease progression and survival are of specific interest. Similar to a diagnostic approach [42], a multivariate binary logistic regression approach with a combination of GM and WM features could be used to predict the survival of more or less than 18 months of patients with ALS [46]. This categorical model achieved an accuracy of 77% on the training data set. However, underperformance on the validation data set suggests overfitting of the model. A deep learning network reached an accuracy of 63% in the prediction of short-, medium-, and long-term survival of patients with ALS based on WM connectivity in terms of FA [43]. Systematic validation studies of such prognostic models on larger multi-site data sets are urgently needed.

### Combinations with Non-Imaging Parameters

Instead of multiparametric MRI analysis, DTI parameters can also be extended by clinical measurements in the feature sets of classification tasks. In categorical survival predictions, such combinations of imaging features with clinical parameters may lead to improvement in the model accuracy compared to predictions solely based on diffusion metrics [43,46]. A study investigating the biomarker value of cortical thickness from structural MRI, functional scores, and neurophysiological parameters revealed different temporal dynamics of the modalities longitudinally which may lead to differences in the sensitivity to disease progression [44]. Differences between neuroimaging, functional rating, and neurophysiological measures in the sensitivity to cerebral progression might imply that by combining MRI, and specifically DTI, with non-imaging modalities, the disease status of patients might be characterized in detail and monitored longitudinally.

As clustering algorithms are frequently applied to the identification of patterns in unlabeled data sets, a multiparametric data set from WM, an oculomotor, and cognitive parameters associated with neuropathological stages of ALS could be analyzed in a data-driven manner without prior inclusion of the patient’s disease status [45]. In this study, hierarchical agglomerative clustering indicated a division of a heterogeneous group of patients with ALS into four clusters, each with similar multimodal parameters. Statistical analysis revealed differences in all parameters across clusters. Patients in one cluster showed the highest FA values and best performance in executive oculomotor tasks and cognitive tests, whereas patients in the most distant cluster showed the lowest FA values, lowest cognitive scores, and worst executive oculomotor performance across all clusters. Thus, the clustering approach showed high congruence of DTI, executive oculomotor function, and neuropsychological performance in patients with ALS. It seemed safe to conclude that the four clusters are in vivo correlates of neuropathological spreading stages. The development of an in vivo staging concept considering different brain function parameters could compensate for each modality’s limitations and lead to an in-depth characterization of patients with ALS. A multivariate DTI staging algorithm based on Bayesian statistics might provide the computational framework for such an approach. Such a classifier was superior to the classical threshold-based method in staging patients with ALS at the individual level [37]. The significant advantage of Bayesian statistics for multimodal issues is the ability to incorporate prior knowledge about the patient into the algorithm directly. With Bayesian statistics, the transition to statements expressing a degree of belief in how likely a specific event is has also proven useful for other research questions in ALS, such as hypothesis testing or complex networks [60,61].

Fluid markers from CSF or plasma are also conceivable combination parameters in comprehensive multimodal models. Neurofilament (NF) concentrations have previously been shown to have prognostic and predictive value [19] that might favor their inclusion together with DTI parameters in models for diagnostic classification or prediction of disease progression. MRI, which is able to regionally map the disease-related stages of ALS in vivo, has a differential part than fluid markers, such as the NF light chain, which correlates with disease progression rate and is negatively associated with survival and thus provides prognostic information [19].

## 4. Longitudinal Monitoring

### 4.1. Study Conceptualization

For longitudinal monitoring and clinical trials, reliable markers sensitive to cerebral progression are needed. Although alterations of the FA in the CST could be monitored longitudinally [11,17] and correlated with the loss of functionality [11,62], some studies reported negative results [63,64]. To obtain sensible and reproducible results in longitudinal DTI studies in ALS, it is essential to establish standards for study conceptualization. The main limitation of longitudinal DTI studies is insufficient sample size leading to insufficient statistical power. Under-powered studies hamper the investigation of the therapeutic effects of DTI [65]. Reports of sample sizes needed for 80% power with 25% treatment effect differ between 263 [17] and 567 subjects [66] per arm. In addition, 128 per arm for 50% treatment effect and 70% power were reported [11]. In all these studies, statistical power and sample size calculations were performed post hoc; therefore, the results might differ from the true power [67]. Monte Carlo simulations can be used in study conceptualization to calculate statistical power and sample size requirements under various study conditions. In addition to sample size, the power of a longitudinal DTI study is influenced by other factors such as the number of visits and time between them, data quality, between-subject variability, class probabilities, and the number of study drop-outs. For different decrease rates of FA in CST, Monte Carlo simulations showed that longitudinal group studies benefit from a second DTI scan at each visit [68]. Specifically, it was revealed that a second scan can reduce the required sample size or that sufficient statistical power could be achieved after shorter time intervals, respectively. The positive effect of repeated scans per visit was particularly pronounced with high measurement noise which is likely to occur with pronounced disease severity.

The scheduling of more than two visits is especially critical to the validity of imaging parameter trend analyses in longitudinal studies with an odd number of visits in total, e.g., one baseline and two follow-up visits [69]. With the simulation of outliers due to noise, it was shown that equidistant time intervals should be avoided to strengthen the trend analysis results in these specific scenarios. Although the study was based on the longitudinal striatum atrophy in T1w MRI data of patients with Huntington’s disease, the methodological limitations of longitudinal trend analysis are also valid for DTI studies in patients with other progressive neurodegenerative conditions, such as ALS.

A reliable assessment of (small) longitudinal WM alterations is essential in ALS for a timely determination of progression rate and enhances the stratification of patients for enrolment in clinical trials. Additionally, studies monitoring asymptomatic carriers of ALS-associated gene mutations for possible conversion to symptomatic disease might benefit from optimized study protocols to detect even subclinical cerebral alterations. For the conceptualization of clinical neuroimaging studies, considering sample numbers, measurement noise, and visit scheduling, simulations have proven to be useful [68,69]. In order to minimize the stress on patients and save time and money, meticulous planning of neuroimaging scans in advance may prevent the generation of incorrect findings.

### 4.2. Influences of Aging

In longitudinal studies, it is necessary to be aware that diffusion metrics are subject to physiological aging effects [70,71] and to what extent aging-associated alterations can be expected during the study period. To distinguish the proportion of physiological aging effects from neuropathological changes at the group level, it is recommended to longitudinally assess healthy control subjects according to the same study protocol as patients with ALS in prospective studies [72]. A tract-specific age correction, adjusted for the age of the study participants, could then be used to computationally eliminate the covariate age in the diffusion metrics, as in cross-sectional studies [73].

The modeling of the complex trajectories of healthy brain aging with ML approaches based on neuroimaging data has been established for clinical questions in recent years [74,75]. Such a brain age prediction is also possible when it is exclusively based on DTI data [76,77]. In principle, age correction of diffusion metrics in healthy study participants could be performed with an algorithmic inversion of brain age prediction based on an artificial neural network [77]. However, in addition to its role as a confounding covariate in a longitudinal setting, age is also a significant risk factor in sporadic ALS [78]. Brain age predictions have already been applied to ALS based on structural MRI, offering insights into a potential brain reserve against behavioral and/or cognitive decline and faster disease progression [79]. Based on these findings, multimodal MRI brain age models might offer promising approaches to investigate other risk factors regarding the personal environment since many lifestyle and biomedical parameters are associated with brain age [80,81,82].

## 5. Limitations

One major limitation of DTI-based ML approaches in ALS is the limited availability of data sets of patients. Longitudinal studies often experience high drop-out rates due to the rapid and often not predictable clinical disease progression. However, the number of data sets available often leads to a low SFR and is crucial in the choice of a classification model. While well-regularized standard ML models, such as SVMs or RFs, might be on the edge of overfitting given the typical sample size in ALS of around 20 to 200, i.e., an order of magnitude 10^1^ to 10^2^, deep learning models require several orders of magnitude in sample size more to perform optimally [1]. On the one hand, the risk of overfitting in standard ML models can be reduced with *a priori* feature selection based on neuroanatomical considerations. On the other hand, limiting features only to DTI and T1w metrics of specific neuroanatomical regions also appears to restrict the discriminating power of standard ML models, as different ML approaches with similar preselected multiparametric MRI features led to similar accuracies in diagnostic classifications.

In the context of the performance of DTI-based ML models, the physiological inter-subject variability of diffusion metrics should be discussed in addition to the limited sample sizes in ALS. Many lifestyle factors such as smoking [83], alcohol consumption [84], or sleep duration [85], have been reported to result in regional WM alterations. A more detailed description of healthy controls with respect to certain lifestyle circumstances might allow a better assessment of diffusion metrics and thus possibly a more accurate separation of patients and controls by adding confounding lifestyle parameters in ML models.

## 6. Further Perspectives

**Figure 3:**
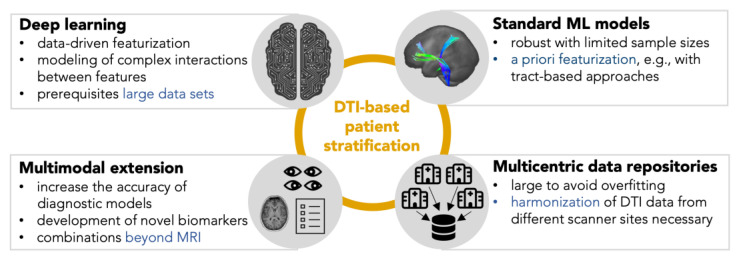
Strategies for the implementation of machine learning models on diffusion tensor imaging (DTI) data in amyotrophic lateral sclerosis. For several clinical questions, standard machine learning algorithms can provide reliable results even with limited sample sizes. Validating models with an independent test sample is essential for good modeling practice. Deep learning can be implemented with well-curated international data repositories. In addition to more complex algorithms, augmenting DTI features with other magnetic resonance imaging (MRI) parameters or neurophysiological measures can also add value in acquiring knowledge about the progression of the disease.

For the implementation of ML in ALS research, different perspectives arise, as shown in Figure 3. Most DTI-based ML publications in ALS focused on diagnostic classifications, i.e., patient vs. healthy control scenarios. Apart from the limited accuracy, the practical value of diagnostic classification models might be limited since the ground truth of the classifications is a diagnosis based on clinical criteria. The focus should, therefore, turn toward models representing the cerebral status or addressing the heterogeneity of ALS by phenotype classifications or individual disease trajectory predictions. Clustering as a method of unsupervised ML is suitable for the identification of unbiased subgroups in data. The required sample size to detect subgrouping with sufficient power is about 20 to 30 per expected cluster [86]; sample sizes that are realistically achievable in ALS research, even in monocenter settings.

To advance the power to discriminate patients from controls and thus contribute truly to faster diagnosis in ALS, normative deep learning models should find their way into ALS imaging. Deep learning models can encode meaningful representations of brain function in a data-driven manner and determine whether an unknown data set belongs to a group of healthy controls in normative modeling [87]. To advance the use of deep learning in ALS in the future, prospective multicenter neuroimaging studies and international repositories (such as those provided by the Neuroimaging Society in ALS (NiSALS) [15,88,89]) are needed. Thereby, the focus should also be put on longitudinal data collection with standardized protocols. DTI data sets from different centers and scanners should then be harmonized prior to model training, as differences in diffusion metrics may occur from different scanner and data acquisition factors [90]. It was shown that inter-scanner differences could be compensated with harmonization approaches, whereas biological inter-subject differences were preserved in healthy controls, as well as in patients with pathologically altered diffusion properties [15]. Due to inter-scanner differences, ML models need to be validated between centers and scanners for prospective clinical use. Novel techniques, such as deep and transfer learning [91] and few-shot learning [92], offer new possibilities and applications in the characterization and monitoring of ALS patients. Approaches with federated deep learning in multicenter design can be regarded as a perspective.

Diffusion kurtosis imaging (DKI) is a promising tool to be used as a biomarker in neurodegenerative diseases and, thus, also in ALS [93]. The DKI metrics alterations indicate decreased microstructural complexity in ALS, involving motor regions, extramotor regions, and callosal regions at early stage ALS [94,95]. Thus, DKI metrics can serve as potential biomarkers for assessing disease severity [96]. Multiparametric MRI assessments in patients with ALS are promising for enhanced individual phenotyping and may help with the stratification of therapeutic trials, provided that robust ML models will be used. Combining DTI with modalities beyond MRI, such as radioligand imaging with positron emission tomography or measures of neurophysiology [97,98], is a helpful technical approach to deep phenotyping of a given patient’s disease status. Because such approaches will continue to become more important for in vivo ALS staging systems in the future, patients with ALS should be evaluated in studies by use of a variety of clinical and cognitive parameters in addition to MRI [88,99].

An integration of DTI into clinical trials spans two different aspects: stratifying patients into prognostic groups and providing reliable markers sensitive to cerebral progression. DTI-based ML models that predict progression rate and assess phenotype might be appropriate for stratifying patients for clinical trial enrollment. For the establishment of longitudinal DTI biomarkers in clinical trials, the standardization and optimization of study protocols are essential to enhance longitudinal sensitivity and reliability. The use of DTI as a longitudinal monitoring tool could allow objective monitoring of cerebral progression and might be considered to be an endpoint in clinical trials.

## 7. Summary

The current state of research demonstrates the enormous academic and clinical potential of ML models in the development of DTI-based neuroimaging biomarkers in ALS. A more accurate assessment of cerebral changes seems possible with multiparametric MRI feature sets than with DTI metrics alone. To capture the full spectrum of pathological signatures in ALS, combining DTI with other modalities, e.g., neurophysiology, is promising to guide individualized ML-based patient characterizations. As multimodal integration of diffusion-weighted MRI techniques will be an essential element in the development of neuroimaging biomarkers in ALS, future research should focus on establishing standardized protocols for full patient characterization and multicenter transnational collaborations. Large, well-characterized cohorts enable a profound performance of ML algorithms and, therefore, gain new insights into the complex interplay between neuroimaging and other clinical measures to characterize patients with ALS and its phenotypes.

## Figures and Tables

**Table 1:** Diffusion tensor imaging (DTI)-based machine learning models in amyotrophic lateral sclerosis (ALS). AD—axial diffusivity, CC—corpus callosum, CST—corticospinal tract, FA—fractional anisotropy, FAS—flail arm syndrome, HC—healthy controls, LMND—lower motor neuron disease, MD—mean diffusivity, PBP—progressive bulbar palsy, PLS—primary lateral sclerosis, PUMN—pure upper motor neuron, RD—radial diffusivity, RF—random forest, SVM—support vector machine.

Study	Algorithm	Task	Features	Sample Size	Model Validation	Accuracy
Chen et al. [29]	SVM	Diagnostic prediction	FA values of all white matter voxels	22 patients with ALS; 26 HC	Leave-one-out cross-validation	83%
Ferraro et al. [31]	SVM	Diagnostic prediction	FA, MD, AD, and RD of CST and motor callosal tracts	123 patients with ALS; 44 patients with PUMN; 20 ALS-mimics; 78 HC	Independent test sample	78%
Kocar et al. [32]	SVM	Diagnostic prediction	FA of CST, corticopontine tract, corticorubral tract, corticostriatal pathway, proximal perforant path, CC area II, and CC area III	98 patients with ALS; 98 HC	Leave-one-out cross-validation	66%
Münch et al. [33]	SVM	Diagnostic prediction	FA of CC area I–III	432 patients with ALS; 55 patients with PLS; 45 patients with FAS; 22 patients with PBP; 21 patients with LMND; 112 HC	Independent test sample	65%
Li et al. [34]	SVM	Prediction of progression	White matter network matrices	73 patients with ALS	Nested cross-validation	85%
Sarica et al. [30]	RF	Diagnostic prediction	FA, MD, AD, and RD of CST voxels	24 patients with ALS; 24 HC	5-fold cross-validation	80%
Fratello et al. [35]	Multi-view models with RF	Diagnostic prediction	whole-brain FA maps	41 patients with ALS; 37 patients with Parkinson’s disease; 43 HC	n/a	58%
Gabel et al. [36]	Event-based modeling	Ordering of events, i.e., regional involvement	FA of CST (inferior/middle/superior), CC (genu/body/splenium), cingulum (dorsal section), superior longitudinal fasciculus, inferior longitudinal fasciculus, inferior fronto-occipital fasciculus, and uncinate fasciculus	154 patients with ALS; 128 HC	Cross-validation	n/a
Behler et al. [37]	Multivariate Bayesian classification	Cerebral stage prediction	FA of CST, corticopontine tract, corticorubral tract, corticostriatal pathway, and proximal perforant path	325 patients with ALS; 130 HC	Comparison to threshold-based DTI staging	n/a
Bede et al. [38]	Multilayer perceptron	Diagnostic prediction	FA, MD, AD, and RD of 30 white matter regions	214 patients with ALS; 37 patients with a non-ALS neurodegenerative diagnosis; 127 HC	Independent test sample	79%

**Table 2:** Multimodal diffusion tensor imaging (DTI)-based machine learning models in amyotrophic lateral sclerosis. AD—axial diffusivity, CC—corpus callosum, CST—corticospinal tract, FA—fractional anisotropy, FAS—flail arm syndrome, HC—healthy controls, LMND—lower motor neuron disease, MD—mean diffusivity, PBP—progressive bulbar palsy, PLS—primary lateral sclerosis, PUMN—pure upper motor neuron, RD—radial diffusivity, RF—random forest, SVM—support vector machine.

Study	Algorithm	Task	Modalities	Sample Size	Model Validation	Accuracy
Ferraro et al. [31]	SVM	Diagnostic prediction	DTI (tract-based diffusion metrics) + T1w MRI (cortical thickness)	123 patients with ALS, 44 patients with PUMN, 20 ALS mimics; 78 HC	Independent test sample	91%
Kocar et al. [32]	SVM	Diagnostic prediction	DTI (tract-based diffusion metrics) + T1w MRI (texture parameters)	98 patients with ALS; 98 HC	Leave-one-out cross-validation	80%
Münch et al. [33]	SVM	Diagnostic prediction	DTI (tract-based diffusion metrics) + T1w MRI (texture parameters)	432 patients with ALS, 55 patients with PLS, 45 patients with FAS, 22 patients with PBP, 21 patients with LMND; 112 HC	Independent test sample	84%
Bede et al. [41]	Canonical discriminant function	Diagnostic prediction	DTI (ROI-based diffusion metrics) + T1w MRI (ROI-based signal intensity + basal ganglia volumetrics)	75 patients with ALS; 75 HC	Independent test sample	90%
Fratello et al. [35]	Multi-view models with RF	Diagnostic prediction	DTI (whole-brain FA maps) + fMRI (whole-brain default-mode networks)	41 patients with ALS, 37 patients with Parkinson’s disease; 43 HC	5-fold cross-validation	67%
Schuster et al. [42]	Binary logistic regression	Diagnostic prediction	DTI (ROI-based diffusion metrics) + T1w MRI (regional grey matter densities)	81 patients with ALS; 66 HC	Independent test sample	78%
Schuster et al. [43]	Binary logistic ridge regression	Survival prediction	DTI (regional diffusion metrics) + T1w MRI (regional cortical thickness)	60 patients with ALS; 69 HC	Independent test sample	58%
Kocar et al. [32]	Multilayer perceptron	Diagnostic prediction	DTI (tract-based diffusion metrics) + T1w MRI (texture parameters)	98 patients with ALS; 98 HC	Independent test sample	72%
Bede et al. [38]	Multilayer perceptron model	Diagnostic prediction	DTI (ROI-based diffusion metrics) + T1w MRI (cerebral volumes + cortical thicknesses)	214 patients with ALS, 37 patients with a non-ALS neurodegenerative diagnosis; 127 HC	Independent test sample	75%
Van der Burgh et al. [44]	Deep learning networks	Survival prediction	DTI (tract-based FA) + T1w MRI (cortical thicknesses + subcortical volumes) + clinical parameters	135 patients with ALS	Independent test sample	84%
Tan et al. [45]	Probabilistic network-based clustering	Divide patients into subgroups of similar neurodegeneration patterns	DTI (white matter connectome FA) + T1w MRI (whole-brain cortical thickness)	488 patients with ALS; 338 HC	Longitudinal subsample	90% in the validation sample
Behler et al. [46]	Hierarchical clustering	Divide patients into subgroups of similar neurodegeneration patterns	DTI (tract-based FA) + video-oculography + cognitive scores	245 patients with ALS	No	n/a

## Data Availability

Data sharing is not applicable.
